# Evaluation of Two Statistical Methods Provides Insights into the Complex Patterns of Alternative Polyadenylation Site Switching

**DOI:** 10.1371/journal.pone.0124324

**Published:** 2015-04-14

**Authors:** Jie Li, Rui Li, Leiming You, Anlong Xu, Yonggui Fu, Shengfeng Huang

**Affiliations:** 1 State Key Laboratory of Biocontrol, Guangdong Province Key Laboratory of Pharmaceutical Functional Genes, College of Life Sciences, Sun Yat-sen University, Guangzhou, People’s Republic of China; 2 Beijing University of Chinese Medicine, Beijing, People’s Republic of China; University of California, Los Angeles, UNITED STATES

## Abstract

Switching between different alternative polyadenylation (APA) sites plays an important role in the fine tuning of gene expression. New technologies for the execution of 3’-end enriched RNA-seq allow genome-wide detection of the genes that exhibit significant APA site switching between different samples. Here, we show that the independence test gives better results than the linear trend test in detecting APA site-switching events. Further examination suggests that the discrepancy between these two statistical methods arises from complex APA site-switching events that cannot be represented by a simple change of average 3’-UTR length. In theory, the linear trend test is only effective in detecting these simple changes. We classify the switching events into four switching patterns: two simple patterns (3’-UTR shortening and lengthening) and two complex patterns. By comparing the results of the two statistical methods, we show that complex patterns account for 1/4 of all observed switching events that happen between normal and cancerous human breast cell lines. Because simple and complex switching patterns may convey different biological meanings, they merit separate study. We therefore propose to combine both the independence test and the linear trend test in practice. First, the independence test should be used to detect APA site switching; second, the linear trend test should be invoked to identify simple switching events; and third, those complex switching events that pass independence testing but fail linear trend testing can be identified.

## Introduction

Polyadenylation refers to the addition of a poly(A) tail to an mRNA molecule; this process is an essential step in the production of mature mRNA, from pre-mRNA, for translation in eukaryotes. Alternative polyadenylation (APA) generates diverse mRNA isoforms and, hence, plays an important role in the fine tuning of gene expression by influencing mRNA stability, translation and translocation in cells via changes of the mRNA secondary structures or interaction with regulatory elements [1–7]. More than half of the human protein-coding genes have multiple APA sites [8], and the dynamic usage of such sites is correlated with cell activation, growth, proliferation and differentiation [9–11]. Currently, several high-throughput RNA-seq technologies for genome-wide APA site profiling exist, including Poly(A) capture [12], DRS [13,14], 3P-Seq [15,16], SAPAS [17,18], PAS-seq [19], Poly(A)-seq [20], 3’READs [21] and 3’T-fill [22]. APA site switching refers to the differential usage of APA sites of a gene between samples of different physiological states. Different APA sites generate different tandem 3’-UTRs of variable length, and the longer and shorter 3’-UTRs usually contain more or fewer *cis*-regulatory elements, respectively, especially microRNA target sites [8,23]. In line with this trend, widespread 3’-UTR shortening has been observed to correlate with elevated cell proliferation or cell transformation [23–25]. Therefore, in early studies, APA site switching was treated as a proxy for measuring changes in the relative expression of short versus long 3’-UTR isoforms [12,15,16,19–21,23,26].

Independence tests have been used to evaluate the significance of switches between two APA sites, the proximal versus the distal [26,27]. However, recent 3’-end enriched RNA-seq projects have revealed that many genes contain more than two APA sites [17,20], and Fu et al. have argued that independence tests are not suitable for detecting switches among more than two APA sites [17]. Indeed, independence tests discard the information about the order of the APA sites and their distance to the coding region, which might reduce the power of the detection. In addition, imprecision in the 3’-terminal mRNA excision could produce adjacent APA sites separated by a very short distance [28]. In this situation, independence tests might produce false results due to the ignorance of distance information.

Linear trend tests have been proposed as an alternative to independence tests [17]. In theory, the linear trend test is insensitive to closely apposed APA sites because it takes into account the ordinal and distance information of the APA sites. However, as a type of linear regression analysis, linear trend analysis is vulnerable to outlier data points. Furthermore, the linear trend calculation requires that the samples have approximately equal variances. Because this requirement is rarely met, linear trend tests can produce false positives. Finally, linear trend testing is only justified under the condition that the differential usage of APA sites can be interpreted as a simple change of the average length of all 3’-UTR isoforms.

Here, we evaluate the performance of the independence test and the linear trend test, as well as study their differences, using real and simulated data. Though the independence test is generally more effective, the linear trend test has its merits in certain situations. We therefore propose to use both methods in the detection of APA site switching, which allows separation of the APA site switching events into four major categories: two simple switching patterns that represent the 3’-UTR shortening and lengthening, and two complex switching patterns that do not significantly alter the average length of all 3’-UTR isoforms. In our real data recorded for normal versus cancerous human breast cell lines, complex patterns account for 1/4 of the genes showing significant APA site switching. Our study not only provides insights into the complex regulatory mechanisms of APA site switching but also suggests a practical statistical method for separating simple and complex APA site switching events.

## Methods

### APA site switching analysis

**Fig 1:**
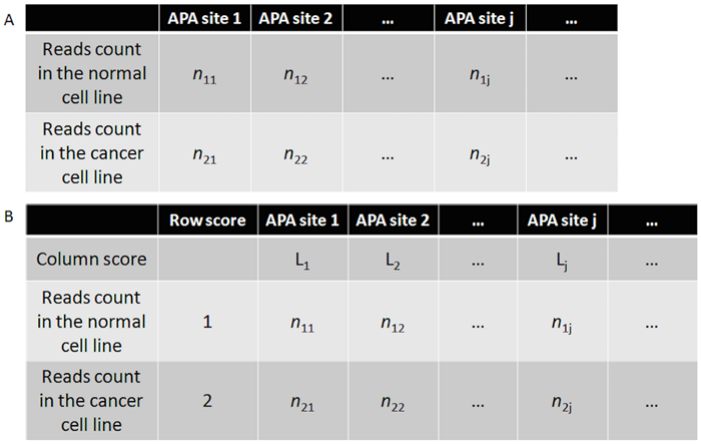
Schematic view of testing tandem APA site switching. (A) Independence testing for the APA site switching. (B) Linear trend testing for the APA site switching. *L*_j_ is the 3’UTR length for the *j*th poly(A) sites and *n*_ij_ is the number of supporting reads for the *j*th poly(A) sites in sample *i*.

The SAPAS data for breast cancer cell line MCF7 and cultured mammary epithelial cell line MCF10A were downloaded from the NCBI Sequence Read Archive (SRA023826). Following filtering and trimming, reads were mapped to the human genome (hg19) using Bowtie [29]; the cleavage sites located within 24 nt were clustered into poly(A) sites as described previously [8]. A tandem file containing the information of gene APA site usage in the two samples was created using the same pipeline and parameters as reported previously [17]. Then, genes that displayed significant APA switching between the cancer and the normal cell lines were identified using two different statistical methods, the linear trend test and the independence test. In the independence test, a column chain table (Fig 1A) is constructed by listing the number of reads for each tandem poly(A) site for each sample in a 2×N table in order (though the actual order does not matter in the independence test), with the two samples as rows (2) and the tandem poly(A) sites as columns (N). The p-value was calculated through Pearson's chi-squared test or Fisher’s exact test using R software. The linear trend test is an analysis for two-way tables with ordered classifications and assigned scores. In the linear trend test, a similar column chain table (Fig 1B) is constructed by listing the number of reads for each tandem poly(A) site for each sample in a 2×N table in order, with the two samples as rows (2) and the tandem poly(A) sites as columns (N). Then the lengths of tandem UTRs denote the scores for the columns; 1 and 2 denote the row scores for sample 1 and sample 2 as dummy variables. Pearson correlation r was calculated by using the number of reads in the table as the values and using the scores for rows and columns as coordinates (Fig 1B). The larger the correlation is in absolute value, the farther the data fall from independence in this linear dimension. In this linear trend test, a statistic M^2^ is calculated using the following formula:$M^{2}=(n-1)r^{2}$, which is approximately chi-squared with one degree of freedom for large samples. The corresponding p-value for the statistic M^2^ was calculated from a chi-squared distribution using R software. The details of two statistical tests are described in Argesti’s Categorical Data Analysis [30]. False discovery rates (FDR) were estimated using the Benjamini-Hochberg process with R software. We developed a Java program called APASAT to conduct these statistical analyses automatically. By default, APASAT performs the chi-squared test and the linear trend test for data points with large sample sizes (> = 30), and it performs Fisher’s exact test for data points of small sample size (<30). Because the linear trend test cannot handle small sample sizes, in this study we only compared the performance of the chi-squared test and the linear trend test using the data points of large-sample size.

### Definition and calculation of the differential APA site usage and the change of normalized average 3’-UTR length

The differential APA site usage for a gene between samples was calculated using the following formula: $DiffInRatio\;=\;\frac{\sum_{i\;=\;1}^{n}\left(\left|{exp1}_{i}/{total}_{exp1}-{exp2}_{i}/{total}_{exp2}\right|\right)}{2}$ (*I* refers to the *i*-th APA site in a gene). The normalized 3’-UTR length for a gene was calculated as the average 3’-UTR length divided by the most distal 3’-UTR length of this gene, and the change of the normalized average 3’-UTR length was calculated as the difference of the normalized 3’-UTR lengths.

### Data simulation

We simulated APA site-switching events involving three poly(A) sites to demonstrate the defects of the linear trend method. To achieve this goal, we generated 3’-UTR lengths and supporting reads for proximal, middle and distal poly(A) sites in two samples. After simulation, APASAT was used to detect the significance of simulated APA switching events by using linear trend tests and chi-squared tests.

### APA switching pattern classification

Using the supporting reads for each poly(A) site divided by the sum of supporting reads for all poly(A) sites in a gene, one can calculate the usage ratios of each poly(A) site in that gene. Then, the usage ratio change can be calculated by subtracting the usage ratio in sample 1 from that in sample 2. In this scenario, a negative number indicates a change “down” and a positive number indicates a change “up”. The absolute value reflects the amplitude of the ratio change: a greater absolute value corresponds to more poly(A) site usage switching. Neighboring sites with the same “up” or “down” trends can be considered single sites, and those whose absolute values are close to zero can be merged with neighboring sites, depending on the situation. This procedure is similar to that used in a change-point model [27] but lacks the limitation that only one change point is allowed. Using this procedure simplifies the situation for genes with many sites and allows most of them to be modeled. Finally, the sites can be divided into four groups based on the number of change points: the “up-down” type (/\), the “down-up” type (\/), the “up-down-up” type (/\/) and the “down-up-down” type (\/\). Note that there can be even more complex regulation patterns when there are many APA sites involved, but their occurrence rates are much lower, and the possible patterns are often able to be approximated by those defined above. Therefore, for brevity and without losing generality; here, we do not explicitly study those even more complex patterns.

## Results

### Performance of the two statistical methods on real data

**Fig 2:**
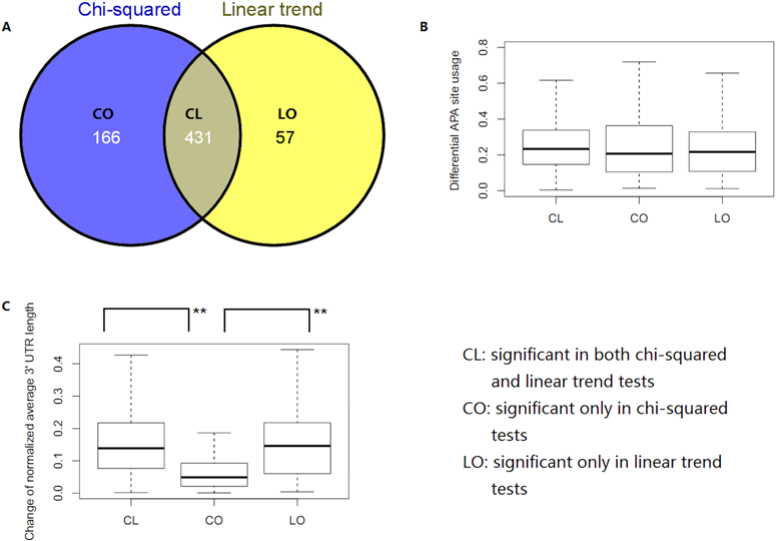
Performance of the two statistical methods on real data from the cultured mammary epithelial cell line MCF10A and the breast cancer cell line MCF7. (A) Venn diagram of APA site-switching genes detected by the chi-squared method and linear trend method between MCF10A and MCF7 (FDR<0.01). (B) Comparison of the differential APA site usage of genes between the three groups shown. $DiffInRatio\;=\;\frac{\sum_{i\;=\;1}^{n}\left(\left|{exp1}_{i}/{total}_{exp1}-{exp2}_{i}/{total}_{exp2}\right|\right)}{2}$, where *i* refers to the *i*-th APA site in a gene. (C) Comparison of the change in the normalized average 3’-UTR length of genes between the three groups. The normalized 3’-UTR length for a gene was calculated by dividing the average 3’-UTR length by the most distal 3’-UTR length of this gene, and the change of the normalized average 3’-UTR length was calculated as the difference of normalized 3’-UTR lengths. Asterisks indicate the results of Wilcoxon rank sum test comparisons: (**) P<0.0001.

Using the same pipeline as described previously [17], we identified 3844 genes with tandem poly(A) sites from the SAPAS RNA-seq data for the cultured mammary epithelial cell line MCF10A and the breast cancer cell line MCF7. Chi-squared tests detected 597 genes (FDR<0.01) that showed significant APA site switching between the two cell lines, whereas linear trend tests identified only 489 genes (FDR<0.01, S1 Table). Despite the fact that a total of 431 genes were detected by both statistical methods, 166 genes that were reported by chi-squared tests were missed by linear trend tests, with 105 genes not passing the p-value threshold (0.01) and 61 filtered by FDR (Fig 2A). In contrast, linear trend tests reported 57 genes that failed to pass chi-squared tests, with 7 genes not passing the p-value threshold (0.01) and 50 filtered by FDR (0.01). This analysis suggests that the independence test could be more powerful than the linear trend test.

We were interested in learning the underlying causes of the differences in APA site-switching detection between these two statistical methods. We examined each of the 166 genes specifically reported by the chi-squared tests and found that most of them (156 genes) were found to contain at least three APA sites. We reasoned that these genes were either true positives with complex patterns of APA site switching or false positives caused by either outlier sites or closely apposed sites. To determine which explanation was correct, we removed all potential APA sites that contained less than 5 supporting reads among 125 genes and re-performed chi-squared tests, which showed that 108 genes remained significant. Therefore, outlier data points were not the major cause of increased detection of site-switching events. We then looked for close, adjacent APA sites (within 40 nt) and found them in 26 genes. These sites were merged, and the chi-squared tests were re-performed, which showed that 23 of 26 genes still remained significant. Even when the 40 nt cutoff was relaxed to 80 nt, we obtained similar results. Therefore, closely apposed sites were also not the major cause. Taken together, most of these 166 genes specifically reported by the chi-squared tests should be true positives rather than false positives. On the other side, of the 57 significant genes reported by only the linear trend test, 50 were actually reported significant (*p*<0.01) by the chi-squared test and merely filtered by the FDR (cutoff = 0.01). This analysis demonstrates that independence tests have better performance than linear trend tests in APA site-switching detection.

### APA site switching is more complex than the average length change of the 3’-UTR

**Fig 3:**
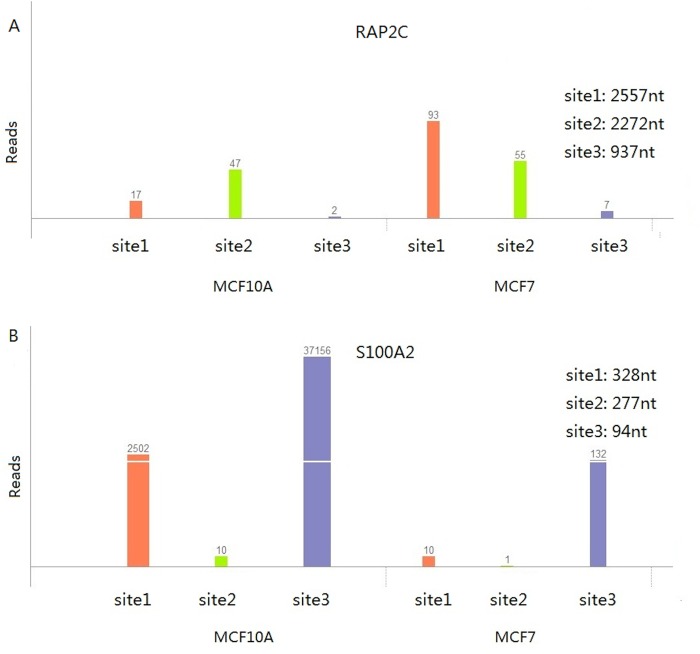
Two example genes showing different APA site-switching patterns between MCF10A and MCF7. Both patterns show little change in the average 3’-UTR length. (A) Ras-Related Protein Rap-2c (RAP2C, uc004ewo.2). (B) S100 Calcium Binding Protein A2 (S100A2, uc001fcb.2). Both genes contain three APA sites, called site1, site2 and site3. The y-axis shows the supporting reads for each site in MCF10A and MCF7 cell lines. The distances from the stop codon to each APA site are displayed on the top right.

To detect length changes of 3’-UTR, the linear trend test can be an effective method. However, in theory, when there are more than two APA sites for a gene, there can be complex APA site switching patterns that cannot be accounted for simply by a change in 3’-UTR length. Indeed, as shown in the analyses above, chi-squared tests detected up to 166 genes that exhibited significant APA site switching between the epithelial cell line MCF10A and its cancer cell line MCF7 but showed no significant 3’-UTR average length changes. To further understand this result, we first divided the significant genes into three groups, as shown in Fig 2A: 431 genes were significant in both the chi-squared and linear trend tests (CL), 166 genes were significant only in the chi-squared tests (CO) and 57 genes were significant only in the linear trend tests (LO). Then, we performed Wilcoxon rank sum tests to evaluate the differences between these groups. The tests showed that there was no significant difference in differential APA site usage among the three groups (Fig 2B), but the change of normalized 3’-UTR length was significantly lower in the CO group (*p* = 1.31e-22, Fig 2C). This finding indicates that the 3’-UTR in the CO group exhibited little length change. In other words, if we assume that chi-squared tests identify all APA switching genes and linear trend tests identify all 3’-UTR-length-changing genes, we can say that in our real case (MCF10A versus MCF7) more than 25% of the APA switching genes showed more complex changing patterns than a simple shortening or lengthening of the average length of all 3’-UTR isoforms. For example, RAP2C is a member of the Ras-related protein subfamily of the Ras GTPase superfamily. Members of this subfamily are small GTPases that act as molecular switches to regulate cellular proliferation, differentiation, and apoptosis [31]. In our real data (MCF10A versus MCF7), this gene has three detectable APA sites and underwent significant APA switching between MCF10A and MCF7 without displaying a significant change in the average 3’-UTR length (*p*<1.6E-5 for chi-squared and *p*<0.17 for linear trend; Fig 3A). More examples are listed in S2 Table and can be visualized on our website, APASdb [32].

The real examples mentioned above may not be straightforward enough to reveal the complex APA switching patterns. We therefore simulated an extreme but typical example: a gene with three APA sites, called the proximal, middle and distal sites, with distances from the stop codon of 1000 nt, 2500 nt, and 4000 nt, respectively. When the proximal and distal sites have equal expression, the average 3’UTR length of this gene will not change at all, regardless of the significant of the middle site change expression level between samples. As a result, the linear trend test failed to detect the APA site switching in this gene (Table 1). The RAP2C example represents exactly this type of situation: the average 3’-UTR length of the proximal and distal APA sites is very close to the length of the middle APA site (Fig 3A). We also provide other simulated examples to show how the APA site distance (Table 2) and the complex changes of expression levels (Table 3) influence the performance of linear trend tests. In our real data (MCF10A versus MCF7), gene S100A2 exhibited a complex APA site-switching pattern similar to the situation described in Table 3 (Fig 3B). This gene is a modulator against excess calcium accumulation in normal human mammary epithelial cells and may play a role in suppressing tumor cell growth. Note that, although we showed only three-APA-site examples here, we could expect that for genes with more than three APA sites, the resulting switching patterns would be even more likely to be missed by the linear trend test. In summary, the linear trend test cannot identify switching events involving complex regulation on more than two APA sites.

**Table 1:** APA site-switching tests on simulated genes whose average 3’UTR lengths for the proximal and distal sites are equal to the 3’UTR length for the middle site.

Sample 1	Sample 2	P-value for linear trend	P-value for chi-squared
100, 100, 100	100,100,100,	1	1
100, 100, 100	100,500,100,	1	2.67E-28
100, 100, 100	100, 1000, 100	1	2.37E-67
100, 100, 100	10, 100, 10	1	2.21E-19
100,100,100	1000,100,1000	1	1.22E-61

**Table 2:** APA site-switching tests on simulated genes whose average 3’UTR length for the proximal and distal sites is a certain distance from the 3’UTR length for the middle site.

Distances of poly(A) sites from stop codon	P-value for linear trend	P-value for chi-squared
1000, 2000, 4000	0.004866	2.67E-28
1000, 2300, 4000	0.247235	2.67E-28
1000, 2500, 4000	1	2.67E-28
1000, 2700, 4000	0.247235	2.67E-28
1000, 3000, 4000	0.004866	2.67E-28

*The supporting reads for each poly(A) site in the two samples are 100, 100, 100 and 100, 500, 100, respectively.

**Table 3:** APA site-switching tests on simulated genes whose APA site expression profile changed significantly without causing change of the 3’-UTR length.

Sample 1	Sample 2	P-value for linear trend	P-value for chi-squared
100, 100, 100	10, 960, 200	0.999999	1.39E-97
100, 100, 100	100, 600, 200	0.999999	6.37E-27
100, 100, 100	100, 1100, 300	0.999999	7.67E-53
100, 100, 100	100, 4600, 1000	0.999999	5.14E-212
100, 100, 100	50, 300, 100	1	2.49E-20

*The distances of the poly(A) sites from the stop codon are 1000 nt, 2000 nt, and 4000 nt, respectively.

### Classification of APA site switching patterns

**Fig 4:**
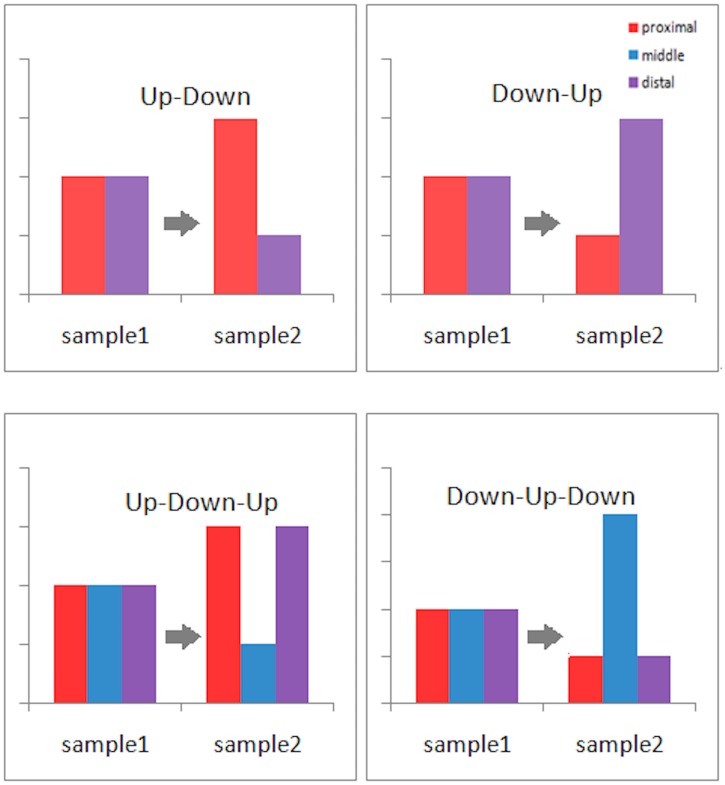
Simplified view of the classification scheme for the APA site-switching patterns based on the site usage ratio change. (1) The “up-down” type (/\) that refers to 3’-UTR shortening, (2) the “down-up” type (\/) that refers to 3’-UTR lengthening, (3) the “up-down-up” type (/\/) that refers to the increasing usage of both the proximal and distal APA sites together with the decreasing usage of middle APA sites, and (4) the “down-up-down” type (\/\) that refers to the decreasing usage of both the proximal and distal APA sites together with the increasing usage of the middle APA sites.

**Fig 5:**
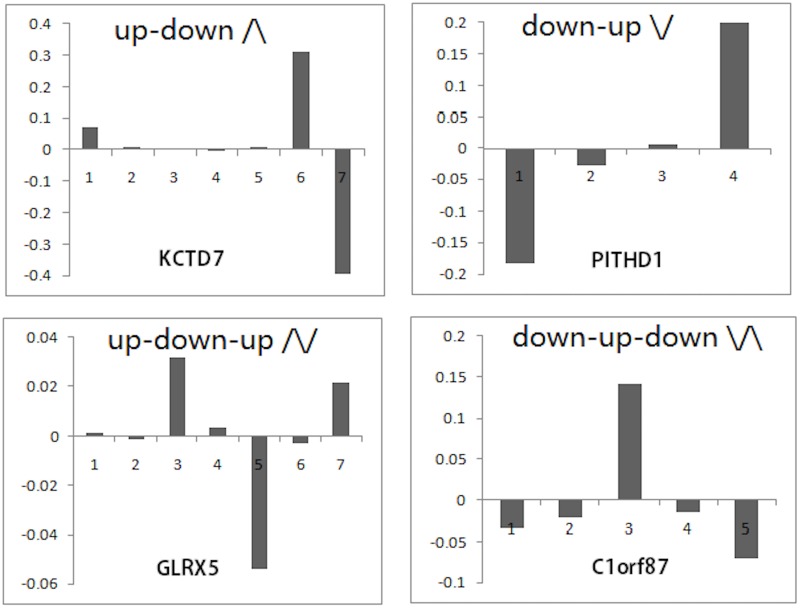
Examples of classification of APA site-switching patterns, based on the site usage ratio change between MCF10A and MCF7. The x-axis represents the APA site, and the y-axis is the change of APA site usage ratio.

Based on the above findings, we classified the APA site-switching events into four basic patterns (Figs 4 and 5): (1) the “up-down” type (/\), which refers to 3’-UTR shortening; (2) the “down-up” type (\/), which refers to 3’-UTR lengthening; (3) the “up-down-up” type (/\/), which refers to the increasing usage of both the proximal and distal APA sites together with the decreasing usage of middle APA sites; and (4) the “down-up-down” type (\/\), which refers to the decreasing usage of both the proximal and distal APA sites together with the increasing usage of the middle APA sites. The first two patterns can be considered to represent simple regulation, and the latter two patterns can be considered to represent complex regulation. There can be more complex regulation patterns, but we can safely omit them here without sacrificing the generality of our study (see Methods for details). In our real data (MCF10A versus MCF7), 431 genes were identified as undergoing significant APA site switching by both chi-squared tests and linear trend tests, of which 371 were classified as /\-type, 48 as \/-type, 5 as /\/-type, and 7 as \/\-type. Of the 60 genes whose chi-squared test p-values were less than 0.01 and whose linear trend test p-values were higher than 0.1, 20 were classified as /\-type, 7 as \/-type, 11 as /\/-type, and 22 as \/\-type. From these numbers, we concluded that most of the APA site switching genes in our real data were subject to simple regulation, and at least 10% of them ((5+7+11+22)/(431+60) = 0.092) were subject to complex regulation. Of those genes undergoing complex APA site-switching regulation, most (33/45) failed to be identified by the linear trend test.

### A combined statistical method and the analyses of other data sets

Based on the above analyses, we propose to use both the independence test and the linear trend test for detection of APA site-switching events. The genes identified by the independence test can be considered to have undergone significant APA site switching, either in simple or complex patterns; in contrast, the genes detected by the linear trend test can be thought of as the genes that have undergone simple switching patterns. Those genes that pass the independence test but fail the linear trend test very likely experience complex regulation. However, those genes that are identified as being marginally significant by only one method should be carefully double-checked before being classified. We have developed a small program called the APA switching analysis toolkit (APASAT) to facilitate the implementation of these two statistical methods in genome-wide detection of APA site-switching genes. Using APASAT, we analyzed our other data sets, including cancer cell line MB231 versus normal cell line MCF7 and different zebrafish developmental stages (available in our public database APASdb [32]: http://mosas.sysu.edu.cn/UTR/). Analyses of these data sets led to the same conclusions as did the analyses above.

## Discussions and Conclusions

Our analyses indicate that the independence test generally outperforms the linear trend test. Closely apposed APA sites and outlier APA sites, which are predicted to confuse the independence test in theory, have little effect on the accuracy of the test in real applications. The linear trend test fails to detect complex APA site-switching events because it assumes that significant switching events should cause changes of the average length of all 3’-UTR isoforms. Linear trend is also insensitive to closely apposed APA sites, but this is not always desirable because a 20-80-bp sequence between two APA sites may be long enough to contain regulatory motifs. The linear trend test has difficulty handling APA sites in introns, internal exons and non-coding genes because there is a problem with quantifying the distance from APA sites to the stop codon in these situations [33,34]. Linear trend testing also cannot handle genes with very low expression levels (i.e., small sample size), whereas Fisher’s exact test (the independence test) can. Despite these disadvantages, the linear trend test still has merits regarding APA site-switching detection, such as its ability to reclaim some marginally significant events and to separate simple and complex switching events. We therefore suggest that at this stage, both statistical methods should be used simultaneously in the detection of APA site switching.

Intuitively, longer 3’-UTR sequences potentially contain more regulatory element binding sites, and *vice versa*; therefore, if APA site switching were always correlated with a change in the average length of 3’-UTR, one might predict that its role is to regulate the number of regulatory element binding sites. However, the fact that in many genes (e.g., approximately 1/4 in our real data), APA site switching results in no significant change of the average 3’-UTR length indicates that regulation of APA site switching is a process with more complex biological effects. Unlike simple shortening and lengthening of the average 3’-UTR length, the functional significance of complex APA site switching is not straightforward and, hence, warrants further investigation. We suspect, however, that there could be at least three motivators for complex switching patterns. First, one may imagine the simple case that a cell needs to generate both short and long 3’-UTR isoforms for a certain gene. Second, the role of *cis*- or *trans*- regulatory elements in APA switching has not been fully understood, so there could be certain complex interactions of *cis*- and *trans*- regulatory elements that might contribute to the complex APA site switching. Third, we should consider the fact that most sequenced samples are mixtures of cells of different cell cycles and physiological states, and these cells each may use totally different APA sites. For example, an early report showed that even in the same cell type, insulin receptor mRNA between growth-arrested cells and proliferating cells used different APA sites [35].

## Supporting Information

S1 Table APA site switching detection results of genes by chi-squared tests and linear trend tests. (TXT)

S2 Table List of genes whose APA site switching is more than average length change of 3’ UTR. (TXT)
